# A comparison of CKD-EPI estimated glomerular filtration rate and measured creatinine clearance in recently admitted critically ill patients with normal plasma creatinine concentrations

**DOI:** 10.1186/1471-2369-14-250

**Published:** 2013-11-13

**Authors:** Andrew A Udy, Fraser JA Morton, Sallyanne Nguyen-Pham, Paul Jarrett, Melissa Lassig-Smith, Janine Stuart, Rachel Dunlop, Therese Starr, Robert J Boots, Jeffrey Lipman

**Affiliations:** 1 Burns, Trauma, and Critical Care Research Centre, The University of Queensland, Royal Brisbane and Womens Hospital, Butterfield Street, Herston 4029, Queensland, Australia; 2 Department of Intensive Care Medicine, Royal Brisbane and Womens Hospital, Level 3 Ned Hanlon Building, Butterfield Street, Herston 4029, Queensland, Australia

## Abstract

**Background:**

The Chronic Kidney Disease Epidemiology Collaboration (CKD-EPI) estimated glomerular filtration rate (eGFR) has been widely integrated into clinical practice. Although useful in screening for CKD, its’ application in critically ill patients with normal plasma creatinine concentrations remains uncertain. The aim of this study was to assess the performance of CKD-EPI eGFR in comparison to creatinine clearance (CL_CR_) in this setting.

**Methods:**

This prospective observational study was performed in a tertiary level, university affiliated intensive care unit (ICU). Study participants had to have an expected ICU length of stay > 24 hours, a plasma creatinine concentration < 121 μmol/L, and no history of prior renal replacement therapy or CKD. CKD-EPI eGFR was compared against 8-hour measured urinary CL_CR_. Data capture occurred within 48 hours of admission.

**Results:**

One hundred and ten patients (n = 110) were enrolled in the study. 63.6% were male, the mean age was 50.9 (16.9) years, 57.3% received invasive mechanical ventilation, and 30% required vasopressor support. The mean CL_CR_ was 125 (45.1) ml/min/1.73 m^2^, compared to a CKD-EPI eGFR of 101 (23.7) ml/min/1.73 m^2^ (*P* < 0.001). Moderate correlation was evident (r = 0.72), although there was significant bias and imprecision (24.4 +/− 32.5 ml/min/1.73 m^2^). In those patients with a CKD-EPI eGFR between 60–119 ml/min/1.73 m^2^ (n = 77), 41.6% displayed augmented renal clearance (CL_CR_ ≥ 130 ml/min/1.73 m^2^), while 7.8% had a CL_CR_ < 60 ml/min/1.73 m^2^.

**Conclusions:**

These data suggest CKD-EPI eGFR and measured CL_CR_ produce significantly disparate results when estimating renal function in this population. Clinicians should consider carefully which value they employ in clinical practice, particularly drug dose modification.

## Background

Accurate assessment of renal function is a priority in the management of critically ill patients. Clinicians regularly utilize such information to help guide drug dosing, optimize fluid, acid–base, and electrolyte management, tailor nutritional requirements, and assess the need for renal replacement therapy (RRT). Rising plasma creatinine (CR) concentrations often trigger clinical interventions, including dose reduction of renally eliminated agents. In contrast, plasma CR concentrations within the reported reference range appear to be less useful. Normal values in the critically ill have been associated with both augmented creatinine clearance (CL_CR_) [1], and occult acute kidney injury (AKI) [2].

Driven primarily by a desire to more effectively monitor and screen for chronic kidney disease (CKD), formulae using simple demographic variables have been developed to estimate the glomerular filtration rate (eGFR). The most commonly applied include the Modification of Diet in Renal Disease (MDRD) [3], and newer CKD Epidemiology Collaboration (CKD-EPI) [4] equations. Their application is based principally on large cohort studies that effectively stratify patients in terms of long-term clinical risk [5,6]. This has led to recommendations for widespread laboratory reporting of eGFR [7,8].

While these initiatives represent key developments in improving the quality of care for patients with CKD, some clinicians have expressed concern about the ubiquitous application of eGFR, particularly in dose modification [9]. Use of formulae to help guide drug dosing represents an attractive approach, although an ability to trigger both dose reduction and escalation is required. Currently there is a paucity of data examining whether eGFR could be used in place of conventional measures for such a purpose, particularly in the critical care environment. The aim of this study was therefore to compare CKD-EPI eGFR with measured urinary CL_CR_, in a cohort of recently admitted critically ill patients with normal plasma creatinine concentrations.

## Methods

### Setting

This study was performed in a tertiary level, university affiliated, metropolitan intensive care unit (ICU), over a two-month period. Enrolment utilized convenience sampling. Ethical approval was obtained from the institutional Human Research Ethics Committee (HREC/09/QRBW/192), with written informed consent obtained from either the patient or their nominated substitute decision-maker.

### Study population

Study participants had to have an anticipated ICU length of stay (LOS) > 24 hours, a plasma CR concentration < 121 μmol/L, and no history of prior renal replacement therapy or CKD. Patients were excluded if: a) either invasive haemodynamic monitoring or an indwelling urinary catheter (IDC) were not employed as part of standard management; b) they were < 18 years of age; c) they were pregnant; d) rhabomyolysis was clinically suspected or the admission plasma creatinine kinase was > 5000 IU/L; or e) they were in the ‘risk’ category or greater for AKI, as defined by the RIFLE criteria [10]. Patients undergoing an operative procedure within 24 hours prior to admission were classified as ‘surgical’. Planned post-operative admissions were considered ‘elective’.

### Interventions

Demographic and illness severity characteristics, including; age, gender, anthropometric measures, diagnosis, and acute physiology and chronic health evaluation (APACHE) II scores were recorded on admission. Modified (excluding the neurological component) sequential organ failure assessment (SOFA) scores, ventilation parameters, 24-hour fluid balance, vasopressor administration, and diuretic use, were recorded prospectively at the time of CL_CR_ assessment. ICU and hospital LOS, and ICU mortality were determined for all patients. Data capture occurred within 48 hours of admission to the ICU, as determined by staff availability and admission time.

An 8-hour measured CL_CR_ was obtained using the following method. Urine was collected via the IDC between midnight and 0800 hrs, following which urinary volume and CR concentration were determined by laboratory analysis. Concurrent plasma CR concentrations were obtained at a point mid-way through the urinary collection, following which CL_CR_ was calculated using the formula listed below. CR measurement in plasma and urine utilised automated analysers employing a modified Jaffe (alkaline picrate) technique, representing an isotope dilution mass spectrometry (IDMS) traceable assay.

As per convention, CL_CR_ values were normalised to a body surface area (BSA) of 1.73 m^2^. The abbreviated 175 MDRD (175 eGFR), CKD-EPI (CKD-EPI eGFR), and Cockcroft-Gault (CG CL_CR_) equations were used to calculate estimates for comparison, as outlined below. Augmented renal clearance (ARC) was defined as a measured 8-hr CL_CR_ ≥ 130 ml/min/1.73 m^2^, given the association with sub-therapeutic drug concentrations, when using standard doses of renally eliminated antibiotics [11,12].

### List of equations employed

$$\mathrm{BSA}=0.007184\times {\left(\mathrm{Ht}\right)}^{0.725}\times {\left(\mathrm{Wt}\right)}^{0.425}$$

$$CL_{\mathrm{CR}}=\left(U_{\mathrm{CR}}\times U_{\mathrm{Vol}}/P_{\mathrm{CR}}\times 480\right)\times 1.73/\mathrm{BSA}$$

$$\begin{array}{l} \mathrm{CG}\;CL_{\mathrm{CR}}=\left[\left(140‒\mathrm{age}\right)\times \mathrm{Wt}\times \left(1.23\;\mathrm{if}\;\mathrm{male},1.04\;\mathrm{if}\;\mathrm{female}\right)\right]\\ \;/P_{\mathrm{CR}}\times 1.73/\mathrm{BSA} \end{array}$$

$$\begin{array}{l} 175\;\mathrm{eGFR}=175\times {\left(P_{\mathrm{CR}}\times 0.0113\right)}^{‒1.154}\times \mathrm{ag}e^{‒0.203}\\ \;\left(\times 0.742\;\mathrm{if}\;\mathrm{female}\right) \end{array}$$

CKD‒EPIeGFR

$$\begin{array}{l} \mathrm{Females},P_{\mathrm{CR}}\;\leq \;62=144\times {\left(P_{\mathrm{CR}}\times 0.0113/0.7\right)}^{‒0.329}\\ \;\times 0.993{\;}^{\mathrm{age}} \end{array}$$

$$\begin{array}{l} \mathrm{Females},P_{\mathrm{CR}}>62=144\times {\left(P_{\mathrm{CR}}\times 0.0113/0.7\right)}^{‒1.209}\\ \;\times 0.993{\;}^{\mathrm{age}} \end{array}$$

$$\mathrm{Males},P_{\mathrm{CR}}\;\leq \;80=141\times {\left(P_{\mathrm{CR}}\times 0.0113/0.9\right)}^{‒0.411}\times 0.993{\;}^{\mathrm{age}}$$

$$\begin{array}{l} \mathrm{Males},P_{\mathrm{CR}}>80=141\times {\left(P_{\mathrm{CR}}\times 0.0113/0.9\right)}^{‒1.209}\\ \;\times 0.993{\;}^{\mathrm{age}} \end{array}$$

Where CL_CR_ = 8-hr Creatinine Clearance (ml/min/1.73 m^2^), U_CR_ = Urinary Creatinine Concentration (μmol/L), U_Vol_ = Urinary volume (ml), P_CR_ = Plasma Creatinine Concentration (μmol/L), BSA = Body Surface Area (m^2^), Ht = Height (cm), Wt = Weight (kg), CG CL_CR_ = Cockcroft-Gault Creatinine Clearance (ml/min/1.73 m^2^), 175 eGFR = Abbreviated Modification of Diet in Renal Disease 175 formula (ml/min/1.73 m^2^), and, CKD-EPI eGFR = Chronic Kidney Disease Epidemiology Collaboration Equation (ml/min/1.73 m^2^), age (in years).

### Statistical analysis

Continuous data are presented as the mean (SD) or median [IQR] depending on adherence to a normal distribution. Normality was assessed by visual inspection, and a one-sample Kolmogorov-Smirnov test. Categorical data are presented as counts (%). Correlations were assessed using a Pearson correlation coefficient (r). Precision and bias were examined using a Bland-Altman plot, with the bias representing the mean difference between each variable, and precision being one SD from the mean. Comparison of continuous data utilized a paired Students *T*-test. A two-sided *P*-value < 0.05 was considered as statistical significance, and all analyses were performed using SPSS version 21 (IBM Corporation, Armonk, New York) and PRISM version 5 (GraphPad Software Inc, La Jolla, California).

## Results

One hundred and ten patients (n = 110) were enrolled in the study, with all participants completing an 8-hr CL_CR_. Demographic, admission, illness severity and outcome data are presented in Table 1. As illustrated, approximately two-thirds of the cohort was male, the patients were relatively young (50.9 (16.9) years), greater than 50% received invasive mechanical ventilation, and about one-third required vasopressor support. Less than 15% were elective cases, with the majority manifesting systemic inflammation, with or without undergoing prior surgery. As per protocol, plasma CR concentrations were within the normal reference range (68.5 (21.8) μmol/L), and did not change significantly in the following 24 hrs (*P* = 0.157), where data were available. The mean 8-hr CL_CR_ was 125 (45.1) ml/min/1.73 m^2^, 48.2% (n = 53) manifested ARC, and 10 (9.1%) had a CL_CR_ < 60 ml/min/1.73 m^2^.

**Table 1:** Demographic, illness severity and treatment data

**Variable**	**N = 110**
Age, years, mean (SD)	50.9 (16.9)
Gender, male/female, n (%)	70 (63.6)/40 (36.4)
Height, m, mean (SD)	1.71 (0.09)
Weight, kg, mean (SD)	80.9 (22.4)
Body surface area, m^2^, mean (SD)	1.92 (0.24)
APACHE II, mean (SD)	16.1 (6.20)
Modified SOFA, median [IQR]	3 [2-5]
Admission type, n (%)	
- Elective	15 (13.6)
- Emergency	33 (30.0)
- Surgical emergency	37 (33.6)
- Trauma	25 (22.7)
Mechanical ventilation, n (%) (n = 108)	63 (57.3)
Intravenous contrast administration, n (%) (n = 109)	30 (27.3)
Frusemide administration, n (%)	13 (11.8)
Mannitol administration, n (%)	4 (3.6)
Vasopressors, n (%)	33 (30.0)
Systemic inflammatory response syndrome, n (%)	95 (86.4)
Plasma creatinine concentration, μmol/L, mean (SD)	68.5 (21.8)
Plasma creatinine concentration + 24 hrs, μmol/L, mean (SD) (n = 80)	63.0 (19.6)
ICU length of stay, days, median [IQR]	4 [2-10]
ICU mortality, n (%)	11 (10)

APACHE-Acute physiology and chronic health evaluation, ICU-Intensive care unit, SOFA-sequential organ failure assessment.

**Figure 1:**
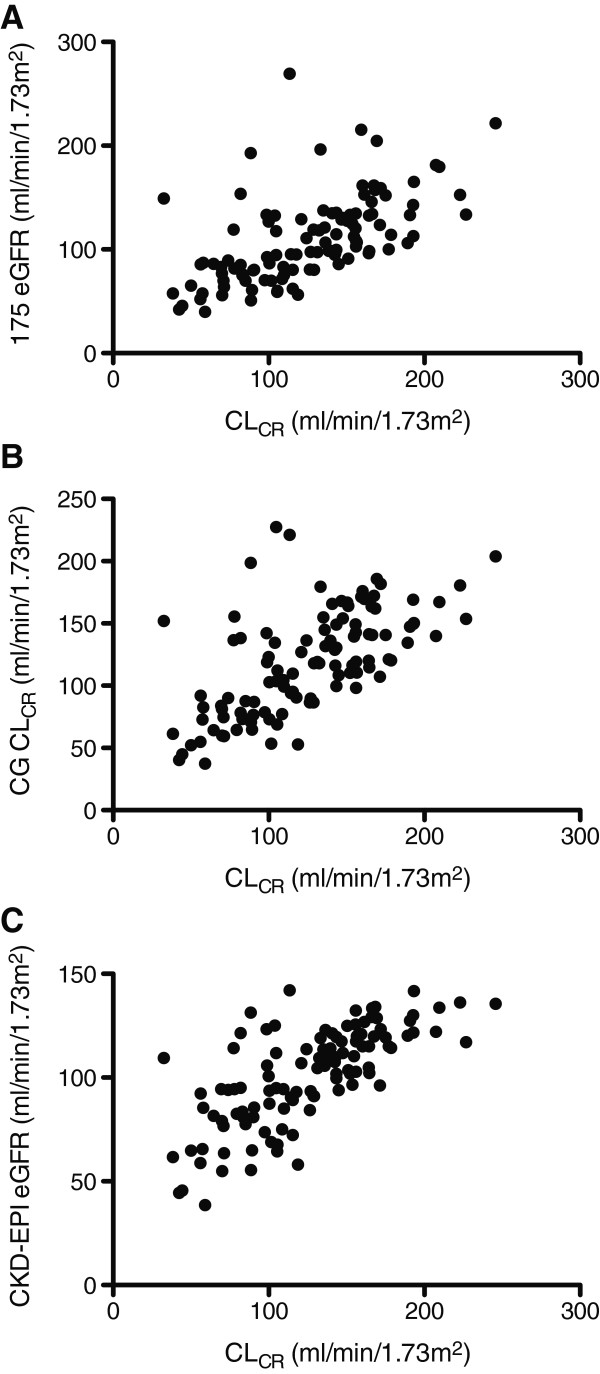
**Scatter graphs of CL**_**CR **_**versus mathematical estimates in all patients.** CL_CR_ on the x-axis compared with 175 eGFR (panel **A**), CG CL_CR_ (panel **B**), and CKD-EPI eGFR (panel **C**), on the y-axis. CL_CR_ = 8-hr Creatinine Clearance, 175 eGFR = Abbreviated Modification of Diet in Renal Disease 175 formula, CG CL_CR_ = Cockcroft-Gault Creatinine Clearance, CKD-EPI eGFR = Chronic Kidney Disease Epidemiology Collaboration Equation.

**Figure 2:**
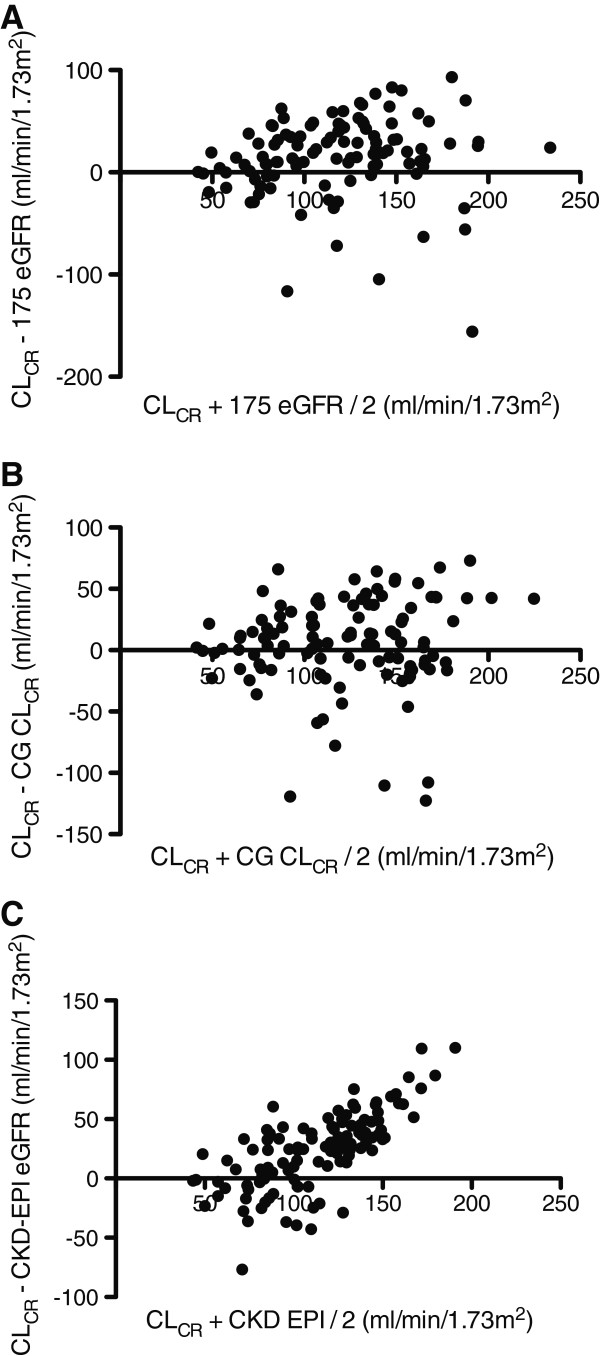
**Bland-Altman plots of CL**_**CR **_**versus mathematical estimates in all patients.** Comparison of the difference between CL_CR_ and 175 eGFR (panel **A**), CG CL_CR_ (panel **B**), and CKD-EPI eGFR (panel **C**) on the y-axis, versus the average value obtained on the x-axis. CL_CR_ = 8-hr Creatinine Clearance, 175 eGFR = Abbreviated Modification of Diet in Renal Disease 175 formula, CG CL_CR_ = Cockcroft-Gault Creatinine Clearance, CKD-EPI eGFR = Chronic Kidney Disease Epidemiology Collaboration Equation.

A comparison of measured 8-hr CL_CR_ and 175 eGFR, CG CL_CR_, and CKD-EPI eGFR in all patients, and each diagnostic category separately, are presented in Table 2. Scatter graphs using all data points are provided in Figure 1. Equivalent Bland-Altman plots are presented in Figure 2. Across all groups, the observed bias is greatest with the CKD-EPI equation. A significant proportional error is also apparent, with higher average values significantly correlated with a larger positive bias (Figure 2C, r = 0.705, *P* < 0.001). This was not evident with either the 175 eGFR (r = 0.102, *P* = 0.289), or CG CL_CR_ (r = 0.103, *P* = 0.285) formulae.

**Table 2:** **Comparison, correlation, bias and precision between measured 8-hr CL**_**CR **_**and mathematical estimates in all patients, and each diagnostic sub-group**

	**Mean (SD)**	**r ( *P *-value)**	**Bias +/− precision**
All patients (n = 110)			
CL_CR_, ml/min/1.73 m^2^	125 (45.1)		
175 eGFR, ml/min/1.73 m^2^	110 (41.6)*	0.600 (<0.001)	15.6 +/− 38.9
CG CL_CR_, ml/min/1.73 m^2^	119 (41.7)	0.638 (<0.001)	6.23 +/− 37.1
CKD-EPI eGFR, ml/min/1.73 m^2^	101 (23.7)*	0.720 (<0.001)	24.4 +/− 32.5
Elective admissions (n = 15)			
CL_CR_, ml/min/1.73 m^2^	118 (27.2)		
175 eGFR, ml/min/1.73 m^2^	115 (51.2)	0.325 (0.237)	2.77 +/− 49.5
CG CL_CR_, ml/min/1.73 m^2^	119 (44.1)	0.531 (0.042)	−1.04 +/− 37.5
CKD-EPI eGFR, ml/min/1.73 m^2^	101 (20.0)**	0.488 (0.065)	17.2 +/− 24.7
Emergency admission (n = 33)			
CL_CR_, ml/min/1.73 m^2^	113 (50.0)		
175 eGFR, ml/min/1.73 m^2^	114 (47.9)	0.624 (<0.001)	−0.77 +/− 42.5
CG CL_CR_, ml/min/1.73 m^2^	123 (49.1)	0.599 (<0.001)	−10.4 +/− 44.3
CKD-EPI eGFR, ml/min/1.73 m^2^	99 (27.6)**	0.692 (<0.001)	13.8 +/− 36.8
Surgical emergency admission (n = 37)			
CL_CR_, ml/min/1.73 m^2^	125 (46.4)		
175 eGFR, ml/min/1.73 m^2^	101 (37.2)*	0.741 (<0.001)	23.7 +/− 31.3
CG CL_CR_, ml/min/1.73 m^2^	108 (37.5)**	0.753 (<0.001)	16.4 +/− 30.6
CKD-EPI eGFR, ml/min/1.73 m^2^	95 (23.7)*	0.779 (<0.001)	29.5 +/− 31.6
Trauma admission (n = 25)			
CL_CR_, ml/min/1.73 m^2^	146 (39.5)		
175 eGFR, ml/min/1.73 m^2^	114 (32.0)*	0.745 (<0.001)	32.7 +/− 26.5
CG CL_CR_, ml/min/1.73 m^2^	129 (33.8)**	0.757 (<0.001)	17.4 +/− 26.1
CKD-EPI eGFR, ml/min/1.73 m^2^	111 (17.4)*	0.772 (<0.001)	35.2 +/− 28.4

* *P* < 0.001, when compared to CL_CR_ ** *P* < 0.05, when compared to CL_CR_.

CL_CR_ = 8-hr Creatinine Clearance, CG CL_CR_ = Cockcroft-Gault Creatinine Clearance, 175 eGFR = Abbreviated Modification of Diet in Renal Disease 175 formula, CKD-EPI eGFR = Chronic Kidney Disease Epidemiology Collaboration Equation, r = Pearson correlation coefficient.

**Figure 3:**
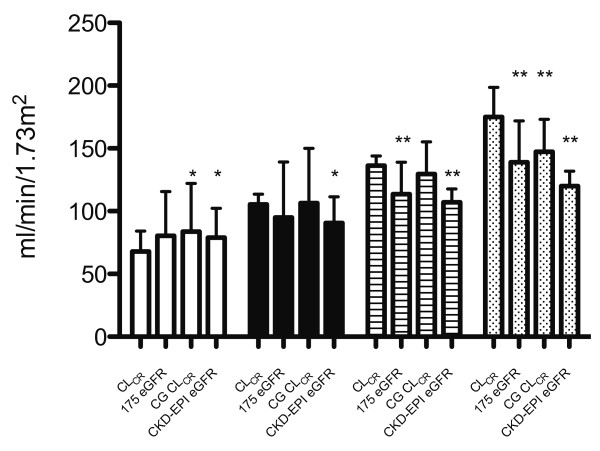
**Comparison of CL**_**CR **_**with mathematical estimates over different ranges.** CL_CR_ compared with 175 eGFR, CG CL_CR_, and CKD-EPI eGFR over different ranges. CL_CR_ < 90 (open), 90–119 (solid), 120–149 (lines), and ≥ 150 (dots) ml/min/1.73 m^2^. * *P* < 0.05, ** *P* < 0.001. CL_CR_ = 8-hr Creatinine Clearance, 175 eGFR = Abbreviated Modification of Diet in Renal Disease 175 formula, CG CL_CR_ = Cockcroft-Gault Creatinine Clearance, CKD-EPI eGFR = Chronic Kidney Disease Epidemiology Collaboration Equation.

8-hr CL_CR_ values were used to categorize patients into four groups; < 90, 90–119, 120–149, and ≥ 150 ml/min/1.73 m^2^. Comparisons with each mathematical estimate are presented in Table 3 and Figure 3. As illustrated, CKD-EPI eGFR was generally higher than CL_CR_ in the lower range (< 90 ml/min/1.73 m^2^), although the opposite was observed at higher values. Correlation was generally poor in each group (Table 3). In those patients with a calculated CKD-EPI eGFR between 60–119 ml/min/1.73 m^2^ (n = 77), 8-hr CL_CR_ values were significantly higher (118 (38.3) vs 96 (16.6) ml/min/1.73 m^2^, *P* < 0.001), 41.6% (n = 32) displayed ARC, and 7.8% (n = 6) had a CL_CR_ < 60 ml/min/1.73 m^2^.

**Table 3:** **Correlation, bias and precision across different ranges of CL**_**CR**_

	**r ( *P *-value)**	**Bias +/− precision (ml/min/1.73 m**^**2**^**)**
CL_CR_ < 90 ml/min/1.73 m^2^ (n = 28)		
175 eGFR	0.223 (0.253)	−12.6 +/− 35.2
CG CL_CR_	0.278 (0.152)	−15.9 +/− 37.2
CKD-EPI eGFR	0.351 (0.067)	−11.1 +/− 23.2
CL_CR_ 90–119 ml/min/1.73 m^2^ (n = 23)		
175 eGFR	0.065 (0.767)	10.5 +/− 44.4
CG CL_CR_	0.066 (0.763)	−0.93 +/− 43.9
CKD-EPI eGFR	−0.067 (0.760)	14.8 +/− 22.8
CL_CR_ 120–149 ml/min/1.73 m^2^ (n = 23)		
175 eGFR	0.047 (0.832)	22.7 +/− 26.1
CG CL_CR_	0.369 (0.083)	6.62 +/− 23.9
CKD-EPI eGFR	0.347 (0.104)	29.2 +/− 10.8
CL_CR_ ≥ 150 ml/min/1.73 m^2^ (n = 36)		
175 eGFR	0.427 (0.009)	36.1 +/− 31.3
CG CL_CR_	0.399 (0.016)	27.8 +/− 27.2
CKD-EPI eGFR	0.460 (0.005)	55.0 +/− 20.9

CL_CR_ = 8-hr Creatinine Clearance, CG CL_CR_ = Cockcroft-Gault Creatinine Clearance, 175 eGFR = Abbreviated Modification of Diet in Renal Disease 175 formula, CKD-EPI eGFR = Chronic Kidney Disease Epidemiology Collaboration Equation, r = Pearson correlation coefficient.

## Discussion

To our knowledge, this is the first report of CKD-EPI eGFR performance in a cohort of Australian patients recently admitted to the ICU. These data demonstrate significant disparity between CKD-EPI eGFR and measured CL_CR_ in patients with normal plasma CR concentrations. Despite an overall reasonable correlation, bias and precision were unacceptable across a range of values. This highlights that clinicians must carefully consider which estimate of renal function they use in clinical decision-making, as these may be very dissimilar. A modest fraction of study participants displayed CL_CR_ measures significantly higher than might be expected, a finding that requires further evaluation.

Albeit the CKD-EPI equation is relatively new in Australian practice, ours is not the only study to explore the use of eGFR formulae in the critically ill. Martin and colleagues examined the utility of MDRD eGFR and CG CL_CR_ in comparison to 8-hr CL_CR_ in a cohort of mainly traumatised patients [13]. CL_CR_ measures were markedly elevated, with significant bias reported with both equations. In ~350 recently admitted patients, Herrera-Gutierrez et al. demonstrated significant bias when comparing CG CL_CR_ to measured values [14]. This was particularly evident in patients with elevated CL_CR_ (≥ 100 ml/min/1.73 m^2^), where CG estimates were markedly lower. Other studies in surgical intensive care [15], and burns injury [16], have reported similar observations.

Hoste and colleagues examined the relationship between 1-hr CL_CR_, CG CL_CR_, and MDRD eGFR in twenty-eight adult patients recently admitted to the ICU [2]. Here, 25% had a 1-hr CL_CR_ < 60 ml/min/1.73 m^2^, despite a normal plasma CR concentration. Even with a lower range of CL_CR_ measures, neither equation was considered specific enough for clinical use [2]. In our study, fewer patients manifest this level of renal impairment (n = 10, 9.1%), limiting any definitive conclusions. However, these patients often (n = 6, 60%) had a normal or near-normal calculated CKD-EPI eGFR (60–119 ml/min/1.73 m^2^).

Baptista and colleagues were the first to explore the role of eGFR in the setting of ARC, comparing CG CL_CR_ and MDRD eGFR with measured CL_CR_ in eighty-six critically ill patients [17]. Calculated values were significantly less than measured CL_CR_, with considerable bias and imprecision. In a retrospective analysis of 390 patients with ARC admitted to a single center, Grootaert and colleagues similarly reported poor agreement between CG CL_CR_, MDRD eGFR and 24-hr measured CL_CR_[18].

Confounding these analyses however, is often the lack of an exogenous marker of GFR. Despite this, markedly elevated renal drug elimination has been noted in many sub-groups of critically ill patients [19], in parallel with higher CL_CR_[20]. Furthermore, recent research suggests elevated CL_CR_ measures (> 130 ml/min/1.73 m^2^) are associated with sub-therapeutic drug concentrations [11,12] and worse clinical outcomes [21], in critically ill patients receiving antimicrobial therapy. While the implications of this phenomenon require substantial validation, the observation that ~40% of patients with a normal or near-normal CKD-EPI eGFR (60–119 ml/min/1.73 m^2^) actually manifest ARC, suggests such thresholds are not simply transferrable to different estimates of renal function.

This realization is consistent with these formulae being developed outside of an ICU environment; generating results that fail to consider the unique characteristics of critical illness [22,23]. Of note, bias appeared to be greatest in emergent surgical and trauma admissions (Table 1), sub-groups where ARC has been previously well documented [24,25]. Recent data from Shimamoto et al. suggests systemic inflammation is a key factor, with increasing SIRS criteria associated with elevated renal vancomycin clearance [26]. This has important ramifications for clinical practice, where use of variable estimates of renal function may result in disparate conclusions [27,28], potentially leading to inadequate drug dosing [29].

We wish to acknowledge the following limitations. This paper reports the findings from a single-center only, and therefore may not be representative of case-mix at other institutions. Despite this, the majority of study participants manifested a systemic inflammatory response; over half received invasive mechanical ventilation; and 30% required vasoactive support. Illness severity scores were moderate, and consistent with tertiary level ICU practice. Our inclusion criteria were designed to select a cohort of patients with normal plasma CR concentrations, as assessing renal function in the context of drug dosing remains challenging in this group. In addition, the CKD-EPI equation is reported to have improved accuracy compared to older eGFR estimates [30], particularly in patients with normal or near-normal renal function [31].

We have employed 8-hr urinary collections, as recommended by prior research [15]. This method is not a gold standard measure of GFR, such that tubular CR secretion, and errors in measurement may have confounded our results. Without employing an exogenous filtration maker (such as inulin), it is impossible to determine which estimate is closer to the ‘true’ filtration rate. As such, use of endogenous CL_CR_ may have resulted in systematically higher values. Despite this, CL_CR_ remains a common modifier of drug dosing in clinical practice, with recent data suggesting important pharmacokinetic [11,12], and clinical [21] implications. Unfortunately no readily accessible, pragmatic, error free measure of GFR is currently available. This analysis principally serves to remind the clinician of the inherent discrepancy between estimates of GFR in the ICU.

## Conclusion

In conclusion, this study has examined CKD-EPI eGFR in comparison to 8-hr measured CL_CR_ in a cohort of recently admitted critically ill patients with normal plasma CR concentrations. Our results suggest poor agreement between these techniques in this population. Whether this represents a true limitation of CKD-EPI eGFR, or an intuitive discrepancy based on the problems with endogenous CL_CR_, remains uncertain. Notwithstanding this, until additional data are available on the utility of CKD-EPI eGFR for drug dose adjustment, particularly in identifying ARC, we would recommend clinicians consider using CL_CR_ for this purpose.

## Abbreviations

AKI: Acute kidney injury; APACHE: Acute physiology and chronic health evaluation; ARC: Augmented renal clearance; CG CLCR: Cockcroft-Gault creatinine clearance; CKD: Chronic kidney disease; CKD-EPI: Chronic Kidney disease epidemiology collaboration; CLCR: Creatinine clearance; eGFR: Estimated glomerular filtration rate; ICU: Intensive care unit; IDC: Indwelling urinary catheter; IDMS: Isotope dilution mass spectrometry; IQR: Inter-quartile range; LOS: Length of stay; MDRD: Modification of diet in renal disease; RIFLE: Risk, injury, failure, loss, end-stage; RRT: Renal replacement therapy; SD: Standard deviation; SOFA: Sequential organ failure assessment.

## Competing interests

All of the authors declare that they have no competing interests in relation to this manuscript.

## Authors’ contributions

AU conceived the study, participated in design and coordination, performed the statistical analysis, and drafted the manuscript. FM and SNP assisted in data collection and database entry. PJ, MLS, JS, RD, and TS participated in data collection. RB and JL provided study oversight, and substantially edited the manuscript. All authors read and approved the final manuscript.

## Pre-publication history

The pre-publication history for this paper can be accessed here:

http://www.biomedcentral.com/1471-2369/14/250/prepub
